# Systematic review of the published data on the worldwide prevalence of John Cunningham virus in patients with multiple sclerosis and neuromyelitis optica

**DOI:** 10.4178/epih.e2018001

**Published:** 2018-01-05

**Authors:** Sonia Patricia Castedo Paz, Luciana Branco, Marina Alves de Camargo Pereira, Caroline Spessotto, Yara Dadalti Fragoso

**Affiliations:** 1 Reference Center for Multiple Sclerosis, Santos, Brazil; 2 Neurology, Neuroscience and Headache Institute, Santos, Brazil; 3 Department of Neurology, Universidade Metropolitana de Santos, Santos, Brazil

**Keywords:** JC virus, Multiple sclerosis, Neuromyelitis optica, Progressive multifocal leukoencephalopathy, Natalizumab

## Abstract

**OBJECTIVES:**

John Cunningham virus (JCV) is a polyoma virus that infects humans, mainly in childhood or adolescence, and presents no symptomatic manifestations. JCV can cause progressive multifocal leukoencephalopathy (PML) in immunosuppressed individuals, including those undergoing treatment for multiple sclerosis (MS) and neuromyelitis optica (NMO). PML is a severe and potentially fatal disease of the brain. The prevalence of JCV antibodies in human serum has been reported to be between 50.0 and 90.0%. The aim of the present study was to review worldwide data on populations of patients with MS and NMO in order to establish the rates of JCV seropositivity in these individuals.

**METHODS:**

The present review followed the PRISMA (Preferred Reporting Items for Systematic Reviews and Meta-Analyses) guidelines and used the following search terms: “JCV” OR “JC virus” AND “multiple sclerosis” OR “MS” OR “NMO” OR “neuromyelitis optica” AND “prevalence.” These terms were searched for both in smaller and in larger clusters of words. The databases searched included PubMed, MEDLINE, SciELO, LILACS, Google Scholar, and Embase.

**RESULTS:**

After the initial selection, 18 papers were included in the review. These articles reported the prevalence of JCV antibodies in the serum of patients with MS or NMO living in 26 countries. The systematic review identified data on 29,319 patients with MS/NMO and found that 57.1% of them (16,730 individuals) were seropositive for the anti-JCV antibody (range, 40.0 to 69.0%).

**CONCLUSIONS:**

The median worldwide prevalence of JCV among adults with MS or NMO was found to be 57.1%.

## INTRODUCTION

John Cunningham virus (JCV) is a ubiquitous polyoma virus that only infects humans [1]. Primary exposure to JCV is asymptomatic and usually occurs in childhood or adolescence, although it may also occur in adulthood [2]. Following infection, JCV can be found in a latent form in several tissues, including the brain [3]. The cell receptors for JCV are an N-linked glycoprotein with a terminal α(2,6)-linked sialic acid, which is present in many types of human cells [4], and the serotoninergic 5HT-2a receptor, which is also present in several types of cells, including those in the central nervous system [5]. Despite the wide distribution of JCV receptors in the body, it has proven to be very difficult to propagate JCV in human cell culture systems [1].

JCV can cause progressive multifocal leukoencephalopathy (PML), a severe disease of the brain resulting from the lytic infection of glial cells in immunosuppressed patients [6]. Management of this potentially lethal infection by rapidly restoring immune function may trigger another dramatic condition known as immune reconstitution inflammatory syndrome (IRIS) [7]. Although acquired immune deficiency syndrome was the main cause of PML for many years, the advent of very potent monoclonal antibody immunological treatments has brought about a new category of patients at risk of PML [8]. In neurology, the use of natalizumab for treating multiple sclerosis (MS) has led both to a remarkably efficient therapy and to a new severe adverse event [9]. The use of anti-CD20 drugs such as rituximab has been reported to be associated with the appearance of PML in rheumatoid arthritis [10]. However, studies of the prevalence of JCV in patients with neuromyelitis optica (NMO) have not been routinely conducted. These patients may undergo treatment with anti-CD20 monoclonal antibodies.

The risk of developing PML in MS can be stratified, understood, and applied to a population of patients [8]. However, it is essential to establish the prevalence of JCV throughout the entire population in order to make better use of recommendations and guidelines on the risk of PML and PML-IRIS. Although data on the prevalence of JCV in the populations of many countries have been published, no systematic review of these data has been carried out. The proportion of the adult population with antibodies to JCV seems to range from 50.0% to 90.0% [11]. The present paper rigorously reviewed the literature on the prevalence of JCV in patients with MS and NMO throughout the world.

## MATERIALS AND METHODS

A rigorous systematic review was carried out using the search terms “JCV” OR “JC virus” AND “multiple sclerosis” OR “MS” OR “NMO” OR “neuromyelitis optica” AND “prevalence.” The terms were searched for both in smaller and in larger clusters of words. The databases searched included PubMed, MEDLINE, SciELO, LILACS, Google Scholar, and Embase, and the review followed the PRISMA (Preferred Reporting Items for Systematic Reviews and Meta-Analyses) guidelines [12]. Only papers containing the search words in the title or abstract were included. References listed in papers selected for full consideration were also used to identify any other potentially relevant articles. Only published articles presenting original data on populations of adult subjects with MS were included. Treatment for MS and its potential influence on the results were not considered in the present review. The methods used to assess JCV in human serum were not standardized. Longitudinal studies conducted to assess seroconversion were not considered, and only a single point in time was included, irrespective of treatment duration. Editorials, abstracts, comments, and communications from conferences were excluded. No meta-analysis of the data was carried out; the results were analyzed primarily in a qualitative manner and are presented in tabular format.

## RESULTS

The initial search returned 1,664 papers, of which 93 were selected by the authors for a full analysis. After discussions, and with all authors’ agreement, 18 articles were included in this review.

In the articles that were analyzed, the presence of JCV in population-based studies was assessed either by means of enzyme-linked immunosorbent assay (ELISA) testing or by second-generation ELISA testing (Focus Diagnostics, Cypress, CA, USA) in serum through [13]. Other methods, such as polymerase chain reaction and urine testing, were only carried out in studies addressing specific patient populations.

**Figure 1.**
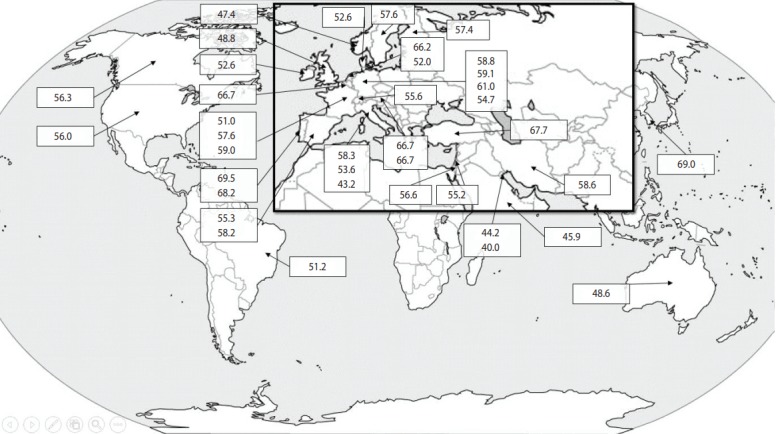
The worldwide prevalence (%) of John Cunningham virus according to population-based studies. Detailed information from each country (including the number of patients enrolled) is presented in Table 1.

The data are summarized in Table 1. Details on the gender, age, and ethnic background of patients were not given in all papers, but there seemed to be an agreement that the prevalence of JCV antibodies increases with age, is higher in men than in women, and is not influenced by ethnicity [14-16]. While some countries only contributed small numbers of subjects, others included thousands. Moreover, while some papers provided information on the prevalence of JCV antibodies in a single country, others reported on several countries and even across continents. The prevalence of positivity for JCV antibodies ranged from 40.0% in Kuwait to 69.5% in Portugal. Interestingly, a second paper from Portugal published 3 years later confirmed this particularly high level of JCV positivity (Table 1). The prevalence of JCV in patients with NMO was assessed in only 1 study, which was conducted in Korea. Positivity for anti-JCV antibodies did not show any continental patterns of distribution, as shown in Table 1 and Figure 1. The systematic review collected data on 29,319 patients with MS/NMO and found that 57.1% of them (16,730 individuals) were seropositive for the anti-JCV antibody.

Although care was taken not to include duplicated data, the present authors cannot be sure that data from the same country in different studies were, indeed, not duplicated. For example, the German population was assessed 4 times and some patients may have been included more than once. Likewise, it is possible that data in the study by Bozic et al. [14] published in 2011 may have been duplicated in later studies [16].

## DISCUSSION

The median worldwide prevalence of JCV among adults with MS or NMO was found to be 58.0%. Previous reports mentioned seropositivity rates between 50.0 and 90.0% [11], but the present review showed that it was between 40.0 and 69.0%. This information is important, since therapy planning for demyelinating diseases of the central nervous system relies more and more on monoclonal antibodies that might, ultimately, be associated with the incidence of PML.

Seropositivity for JCV may be subject to a variety of influences and the values reported by different authors may therefore be somewhat skewed. For example, some large studies did not find any association between JCV positivity and the previous use of natalizumab or other immunosuppressive drugs [15,32]. Other authors reported the contrary, finding that seroconversion rates increased by more than 8.0% per year of use of natalizumab [21,28]. A recent meta-analysis of JCV seroconversion during treatment with natalizumab established that the rate of change of serological status was 10.8% per year [33]. Therefore, the duration of treatment with natalizumab may be a confounding influence on the prevalence of anti-JCV antibodies in patients with MS. Likewise, the method used for assessing antibodies to JCV in serum has improved through second-generation ELISA testing [13], and serum antiJCV titers can now be obtained in routine laboratory reports.

For many countries, there are no published data on JCV prevalence. Thus, the present review cannot be considered representative of the whole world. In Central and South America, the only data available are from Brazil. Furthermore, there is no published information on this subject from any country in Africa.

## Figures and Tables

**Table 1.** Prevalence of JCV antibodies in patients with MS^1^

Country [Ref]	Year	Total population	With JCV (%)
Australia [16]	2014	247	48.6
Austria [15]	2013	666	66.7
Austria [16]	2014	192	66.7
Belgium [16]	2014	206	66.7
Brazil [17]	2013	168	51.2
Canada [18]	2014	4,198	56.3
Denmark [15]	2013	1,402	52.6
Finland [19]	2016	503	57.4
France [20]	2012	361	51.0
France [15]	2013	288	57.6
France [21]	2016	1,259	59.0
Germany [22]	2012	2,782	58.8
Germany [15]	2013	3,415	59.1
Germany [16]	2014	1,736	61.0
Germany [21]	2016	1,921	54.7
Iran [23]	2016	85	58.6
Ireland [16]	2014	100	51.0
Israel [15]	2013	495	56.6
Italy [15]	2013	458	58.3
Italy [24]	2014	97	53.6
Italy [25]	2015	37	43.2
Korea (NMO) [26]	2015	78	69.0
Kuwait [23]	2016	319	44.2
Kuwait [27]	2014	110	40.0
Lebanon [23]	2016	116	55.2
Netherlands [16]	2014	210	66.2
Netherlands [28]	2016	179	52.0
Norway [15]	2013	895	47.4
Portugal [16]	2014	131	69.5
Portugal [29]	2017	371	68.2
Saudi Arabia [23]	2016	61	45.9
Spain [30]	2016	711	55.3
Spain [31]	2017	1,061	58.2
Sweden [15]	2013	2,497	57.6
Switzerland [16]	2014	54	55.6
Turkey [15]	2013	164	67.7
United Kingdom [16]	2014	650	48.8
USA [14]	2011	1,096	56.0
Total	2011/2017	29,319	57.1

JCV, John Cunningham virus; MS, multiple sclerosis; NMO, neuromyelitis optica.

1 Data published in different countries.
